# The effects of contig length and depth on the estimation of SNP frequencies, and the relative abundance of SNPs in protein-coding and non-coding transcripts of tiger salamanders (*Ambystoma tigrinum*)

**DOI:** 10.1186/1471-2164-13-259

**Published:** 2012-06-20

**Authors:** Soo Hyung Eo, J Andrew DeWoody

**Affiliations:** 1 Department of Forestry & Natural Resources, Purdue University, West Lafayette, IN, 47907, USA; 2 Department of Biological Sciences, Purdue University, West Lafayette, IN, 47907, USA; 3 Current address: Department of Zoology, University of Wisconsin, Madison, WI, 53706, USA

**Keywords:** Contig depth, Contig length, Model selection, SNP frequency, Transcriptome, 454 sequencing

## Abstract

**Background:**

Next-generation sequencing methods have contributed to rapid progress in the fields of genomics and population genetics. Using this high-throughput and cost-effective technology, a number of studies have estimated single nucleotide polymorphism (SNP) frequency by calculating the mean number of SNPs per unit sequence length (*e.g.*, mean SNPs/kb). However, both read length and contig depth are highly variable and thus raise doubt about simple methods of SNP frequency estimation.

**Results:**

We used 454 pyrosequencing to identify 2,980 putative SNPs in the eastern tiger salamander (*Ambystoma tigrinum tigrinum*) transcriptome, then constructed analytical models to estimate SNP frequency. The model which considered only contig length (*i.e.*, the method employed in most published papers) was evaluated with very poor likelihood. Our most robust model considered read depth as well as contig length, and was 7.5 × 10^55^ times more likely than the length-only model. Using this novel modeling approach, we estimated SNP frequency in protein-coding (mRNA) and non-coding transcripts (*e.g.*, small RNAs). We found little difference in SNP frequency in the contigs, but we found a trend of a higher frequency of SNPs in long contigs representing non-coding transcripts relative to protein-coding transcripts. These results support the hypothesis that long non-coding transcripts are less conserved than long protein-coding transcripts.

**Conclusions:**

A modeling approach (*i.e.*, using multiple model construction and model selection approaches) can be a powerful tool for identifying selection on specific functional sequence groups by comparing the frequency and distribution of polymorphisms.

## Background

Mutations and subsequent polymorphisms are important not only for identifying influences on genetic disease [1,2], but also for understanding selection, adaptation, and other evolutionary processes [3,4]. While some new mutants have higher fitness than others and therefore are retained by positive selection, most mutations are quickly removed by purifying selection because they are deleterious [5,6]. The intensity of selection may differ across genomic locations (*e.g.*, genic and intergenic regions) and thus nucleotide polymorphisms have different evolutionary impacts depending on their genomic locations. Single nucleotide polymorphism (SNP) profiles can be used to infer the intensity of selection by comparing the frequency and distribution of SNPs of two or more sequence regions, such as genic *vs.* intergenic regions, exonic *vs.* intronic *vs.* untranslated regions, autosomes *vs.* sex chromosomes, and particular gene groups with specific functions [7-9].

To properly compare SNP frequency among groups (*e.g.*, among functional gene groups, among chromosomes, among populations), large samples of similar size are desirable. For example, the International HapMap Consortium [10,11] estimated the frequency and distribution of human SNPs at the genome-wide level; they compared these among three geographically diverse populations by genotyping 90 individuals from each population. To date, such large-scale, systematic evaluations have been limited to human or a few model species *e.g.*, [12].

The advent of high-throughput sequencing technologies (*e.g.*, 454, SOLiD, and Illumina) has contributed to the rapid development of SNPs in non-model but important wild species [7,9,13,14]. In the context of next-generation sequencing, SNP discovery in non-model species is usually achieved by comparing the consensus sequence of a contig with the individual sequences that comprise the contig. However, it is not easy to estimate the frequency of such SNPs because of extreme variation in contig read length and depth; we are more likely to observe SNPs in a 10 kb-long-sequence than in a 1 kb-long-sequence. Furthermore, we have a higher probability of identifying SNPs when we compare contigs comprised of 100 reads than with those comprised of 2 sequence reads. Despite these multiple factors, most next-gen analyses estimate SNP frequency simply by determining the average number of SNPs per unit sequence length (*e.g.*, mean SNP/kb), thus ignoring bias due to contig depth. One of our goals was to determine if such length-only-methods can effectively estimate SNP frequency from next-gen datasets or if more sophisticated approaches are needed.

A second goal was to compare SNP frequencies in protein-coding transcripts *vs.* non-coding transcripts. A “transcriptome” consists of transcripts not only from protein-coding mRNA but also from non-coding RNAs such as ribosomal RNA (rRNA) and transfer RNA (tRNA). In general, non-coding transcripts are expected to be less conserved (*i.e.*, more polymorphic) than protein-coding regions because selection may eliminate deleterious mutations from functional sequences [15,16]. However, recent transcriptome studies have shown that there are many types of non-protein-coding RNAs with functions of biological significance such as chromatin modification and transcriptional regulation [17]. This may imply that at least some non-coding transcripts are as well conserved as protein-coding transcripts. However, (to our knowledge) no study has systematically evaluated SNP frequencies in coding and non-coding transcripts at the transcriptome level. Although there are some reports that long non-protein-coding RNAs in humans and mice have evolved rapidly [18-20], the data are still lacking—especially in non-mammalian species.

One of our ongoing research programs is focused on evolutionary and ecological genomics of tiger salamanders [21-24]. For this research, we sequenced the transcriptome of the eastern tiger salamander (*A. t. tigrinum*) using 454 pyrosequencing and then analyzed the distribution and frequency of SNPs. We studied the influence of contig depth on the estimation of SNP frequencies, and we tested the hypothesis that non-coding transcripts are less conserved than protein-coding transcripts by comparing their SNP frequencies.

## Methods

### Sample preparation and 454 transcriptome sequencing

We captured and sampled seven tiger salamanders at the Purdue Wildlife Area (Indiana, USA) consistent with IACUC 1203000614 and in accordance with guidelines by the American Society of Ichthyologists and Herpetologists and by the American Veterinary Medical Association (June 2007). RNA was extracted from gill, lung, skin and spleen using TRIzol® reagent (Invitrogen). We constructed a total of 19 tissue-specific cDNA libraries from these RNA samples using the ClonTech SMART cDNA synthesis kit, utilizing a modified poly-T primer [25]. Each double-stranded cDNA library was digested with *Sfi*I to remove excess primers and subsequently purified with the QIAquick PCR purification kit (Qiagen). Approximately 5 μg of each cDNA library was sequenced using a 454 Genome Sequencer with FLX Titanium chemistry (454 Life Sciences). Briefly, the cDNA fragments were sheared *via* nebulization, hybridized to DNA capture beads, and amplified by emulsion-based PCR. Molecular barcodes with multiplex identifiers (MIDs, unique 10 bp sequences) were applied to specifically tag each cDNA library in our pooled sequencing run.

### Contig assembly and SNP detection

We pooled all sequences from seven individuals in PCAP [26] and assembled contigs from the entire data set using default settings with the overlap cutoff of 92% identity. The consensus sequence from each of these contigs was used as a reference sequence to which individual sequence reads were aligned using PCAP. First, we identified SNPs only from contigs at least 100 bp in length and with a depth of 10 or more reads. We limited SNP scoring to contigs which have minor allele frequency of 0.01 or at least two minor allele reads. Furthermore, we limited SNPs to regions where 20 bp of high-quality sequence data was present both upstream and downstream of the variable site. We discounted SNPs in homopolymer repeats of > 4 nucleotides, and defined high-quality sites as those with a PCAP base quality score >20. We considered only biallelic SNP substitutions, excluding all indels and triallelic sites. Second (in a separate analysis), we identified SNPs in long contigs (at least 501 bp) [27] using otherwise identical methods. These long contigs are thus a subset of all contigs. Our use of these two datasets (“all” contigs of ≥100 bp and “long” contigs of >500 bp) allowed us to evaluate SNP frequencies in long transcripts.

### Discriminating among protein-coding and non-protein-coding transcripts

We divided all contigs into categories of protein-coding and non-coding transcripts using the program CPC [28]. CPC employs supervised learning algorithms known as support vector machines to discriminate between protein-coding and non-protein-coding transcripts [28,29]. The program assessed the protein-coding potential scores of each contig based on the quality of the predicted open reading frame (ORF) and on the quality of the BLAST hit against UniProt Reference Clusters. CPC was used to determine ORF quality by log-odds score, coverage, and integrity of the ORF whereas BLAST hit quality was determined by BLAST hit number, feature of high-scoring segment pairs (HSP), and HSP frame score among three reading frames. Positive coding potential values reflect protein-coding transcripts whereas negative coding potential values designate non-protein-coding transcripts. Thus, we distinguished protein-coding from non-coding transcripts by considering coding potential scores of a contig (and by checking the score of its reverse complement strand).

### Modeling SNP frequencies and comparisons between protein-coding and non-coding transcripts

Our first goal was to evaluate the effects of contig length and depth on the estimated frequency of SNPs. *A priori*, we predicted that SNP frequency would positively associate with both factors. We employed the information-theoretic approach suggested by Akaike [30] and extended by Burnham and Anderson [31] to evaluate the relative plausibility of models relating contig length and depth to the number of SNPs in contigs. Our second goal was to test the hypothesis that SNP frequency in non-coding transcripts is higher than in protein-coding transcripts. To test this, we included transcript type (protein-coding or non-coding) as a variable in all models. Therefore, the global model consisted of length (LENGTH, the number of bp), depth (DEPTH, the number of aligned sequence reads), and the variable of transcript type (C/NC, coded with 1 for protein-coding transcript and 0 for non-coding transcript) of each contig. We used SAS 9.2 to estimate parameter values.

Four candidate models were fitted using regression models based on a negative binomial distribution. To assess the relative fit of each model, we compared the Akaike’s Information Criteria (AIC), with better fitting models having lower AIC [31]. The relative plausibility or weight of each model was evaluated by measuring Akaike weight (*w*_*i*_). We repeated construction of candidate models and model selection for the number of transitions (*T*_*i*_) and transversions (*T*_*v*_) as the response variables, as well as for the number of SNPs in a contig. For ease of interpretation of the relative effect of each variable, we plotted estimates of the relationships between length or depth of contigs and the number of SNPs found in protein-coding and non-coding transcripts.

## Results

Our 454 pyrosequencing run on the cDNA from seven tiger salamanders yielded a total of 670,408 sequence reads spanning 190.4 Mb. After quality control and other filtering (*e.g.*, repetitive elements, short reads, *etc.*) we assembled the remaining 273,501 high-quality sequences into 51,391 contigs. Among contigs with at least 10 sequence reads, 4,552 and 3,181 contigs were ≥100 and ≥501 bp in length, respectively (Table 1; Additional file 1). In the “all” contig dataset of ≥100-bp contigs, protein-coding transcripts were slightly more rare than non-coding transcripts (2,146 *vs.* 2,406); the pattern was reversed in the “long” dataset of ≥501-bp contigs where protein-coding transcripts were more common (1,762 *vs.* 1,419). A total of 2,980 SNPs that passed our stringent scoring criteria were detected in the “all” contig dataset; of these, 2,515 (84%) were found in the “long” dataset (Additional file 1). The ratio *T*_*i*_/*T*_*v*_ was 1.8 in both datasets. An overview of the assembled contigs and SNPs is presented in Table 1 and our short read sequence data will be deposited at Dryad (http://dx.doi.org/10.5061/dryad.5pd17).

**Table 1:** Summary statistics of assembled contigs (with depth of ≥10 reads) and SNPs

			**Total**	**Protein-coding transcript**	**Non-coding transcript**
*Contigs (with length of 100 bp or longer)*					
	Number of cocntigs		4552	2146	2406
		Mean length (bp)	811	977	664
		Mean depth	28	36	21
	Number of SNPs		2980	1744	1236
		Number of transitions	1917	1139	778
		Number of transversions	1063	605	458
*Contigs (with length of 501 bp or longer)*					
	Number of contigs		3181	1762	1419
		Mean length (bp)	1003	1095	889
		Mean depth	32	39	23
	Number of SNPs		2515	1555	960
		Number of transitions	1603	1009	594
		Number of transversions	912	546	366

**Figure 1:**
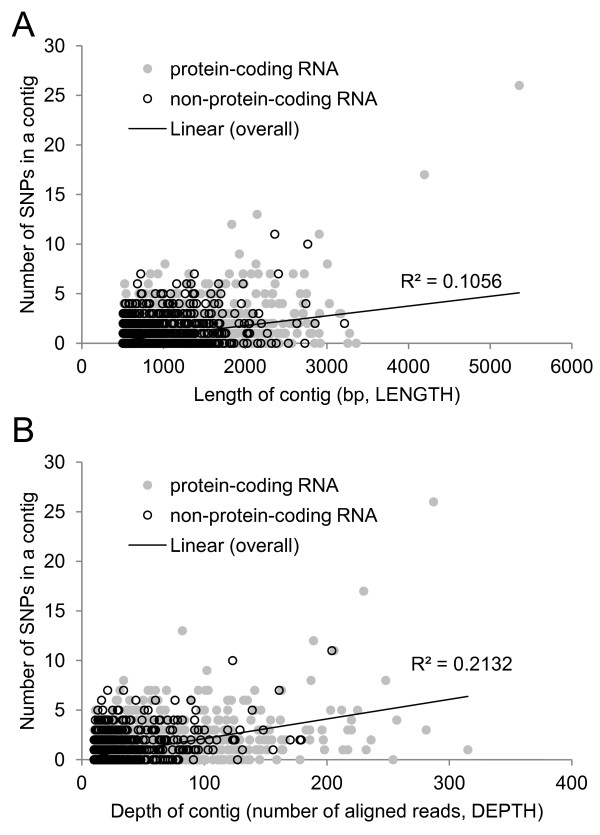
**Correlations between the number of SNPs and both length and depth of contigs.** Positive correlations between the number of SNPs and both **(A)** length and **(B)** depth of contigs with depth of ≥10 reads and length of ≥501 bp. In both cases, *P* < 0.001.

The correlation results were qualitatively similar between the “all” (Additional file 2: Figure S1) and “long” datasets (Figure 1). Here we focus on the long dataset, where the number of SNPs observed in a contig averaged 0.79 and ranged from 0 to 26. Mean contig length in the long dataset was 1,003 bp (range 501 – 5,354 bp) and mean contig depth was 32 reads (range 10–315). We found positive associations between the number of SNPs and contig length (*r* = 0.32; *P* < 0.001; Figure 1A), and between the number of SNPs and contig depth (*r* = 0.46; *P* < 0.001; Figure 1B). These same trends were apparent in both protein-coding (*r* = 0.36 for length *vs.* SNP frequency; *r* = 0.47 for depth *vs.* SNP frequency) and non-coding transcripts (*r* = 0.22 for length *vs.* SNP frequency; *r* = 0.44 for depth *vs.* SNP frequency); all of these associations were significant (*P* < 0.001).

To estimate SNP frequency in the ‘long’ (≥ 501 bp and ≥10 reads) contig dataset, we used the negative binomial distribution to construct four regression models that considered contig length, contig depth, and the category of transcript (protein-coding and non-coding transcript). The most robust model of SNP frequency was the global model that used all of these variables; similarly, the global models were most robust for estimating the frequency of transitions (*T*_*i*_) and transversions (*T*_*v*_) (Table 2). The *w*_*i*_ (relative plausability or weight of each model) of the global models for estimating the frequency of SNPs, *T*_*i*_, and *T*_*v*_, ranged from 0.68 to 0.99. In sharp contrast, those of the commonly-employed “length-but-no-depth” models ranged from 6.7 × 10^-24^ to 1.2 × 10^-56^. Our best-fit model predicting SNP frequency was 7.5 × 10^55^ (= 0.904/(1.2 × 10^-56^)) times more likely than the length-but-no-depth model (Table 2). Note the depth-but-no-length was also a much better model than the commonly employed length-but-no-depth model (Table 2). The “all” contig dataset yielded qualitatively similar results (Additional file 2: Table S1).

**Table 2:** Model selection among candidate models predicting frequencies of SNPs, transition and transversion in contigs

**Model**		**Parameters**^**a**^	**AIC**	***Δ*AIC**^**b**^	**w**_**i**_^**c**^
*for estimating the number of SNPs*					
	M1 (best, full model)	Intercept**, LENGTH*, DEPTH**, C/NC†	7283.4	0.0	0.904
	M2	Intercept**, DEPTH**, C/NC	7287.9	4.5	0.096
	M3	Intercept**, LENGTH**, C/NC	7540.7	257.3	1.2 × 10^-56^
	M4	Intercept**, C/NC**	7748.3	464.9	1.0 × 10^-101^
*for estimating the number of transitions (T*_*i*_*)*					
	M1 (best, full model)	Intercept**, LENGTH†, DEPTH**, C/NC	5682.4	0.0	0.681
	M2	Intercept**, DEPTH**, C/NC	5683.9	1.5	0.319
	M3	Intercept**, LENGTH**, C/NC†	5896.1	213.7	2.7 × 10^-47^
	M4	Intercept**, C/NC**	6061.0	378.5	6.3 × 10^-83^
*for estimating the number of transversions (T*_*v*_*)*						
	M1 (best, full model)	Intercept**, LENGTH**, DEPTH**, C/NC*	4077.1	0.0	0.991	
	M2	Intercept**, DEPTH**, C/NC†	4086.6	9.5	0.009	
	M3	Intercept**, LENGTH**, C/NC	4183.8	106.7	6.7 × 10^-24^	
	M4	Intercept**, C/NC*	4310.4	233.3	2.2 × 10^-51^	

Model selection among candidate regression models using negative binomial distribution predicting the frequency of SNPs, transitions, and transversions in contigs (depth of 10 reads or more; length of 501 bp or longer), using Akaike's Information Criteria (AIC).

^a^ LENGTH and DEPTH are the length and the depth of contigs, respectively, and C/NC is a dummy variable for the type of transcript (protein-coding (coded with 1) vs non-coding transcript (coded with 0). **, *P* < 0.01; *, *P* < 0.05; †, *P* < 0.1) variable in each model.

^b^*Δ*AIC is the difference between the AIC of the best fitting model and that of each model.

^c^*w*_*i*_ is Akaike weight of each model.

**Figure 2:**
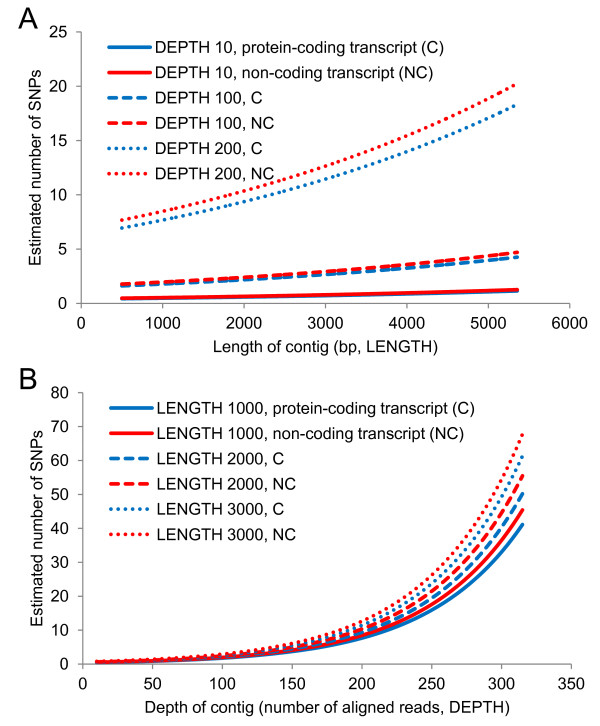
**Estimates of the number of SNPs in transcripts based on length and depth of contigs.** Estimates and comparison of the number of SNPs in protein-coding and non-coding transcripts based on various length and depth of contigs (depth of ≥10 reads; length of ≥501 bp), using best fitting models (Table 3).

Estimation based on the best-fit model (Table 3) indicated that SNP frequency increased with contig length and depth in both protein-coding and non-coding transcripts. Using the long dataset, we estimated a marginally higher frequency of SNPs in non-coding transcripts than in protein-coding transcripts (C/NC coefficient of −0.09; *P* = 0.08) but this trend was not apparent in the “all” contig dataset (C/NC coefficient of 0.04; *P* = 0.43; Additional file 2: Table S2; Additional file 2: Figure S2). From this model, we estimated the number of SNPs in the long dataset as 0.48 (protein-coding) and 0.52 (non-coding) in 1 kb-long contigs with a coverage depth of 10 reads, respectively (Figure 2). Similarly, we predicted 1.78 (protein-coding) and 1.97 (non-coding) SNPs in 1-kb long contigs with a depth of 100 reads, respectively.

**Table 3:** Parameter estimates from best fitting model predicting frequencies of SNPs, transition and transversions in contigs

**Parameter**^**a**^		**Estimate**	**95% confidence limits**		**P**
*for estimating the number of SNPs*					
	Intercept	−0.9828	−1.1137	−0.8520	< 0.0001
	LENGTH	0.0002	0.0000	0.0003	0.0106
	DEPTH	0.0146	0.0127	0.0165	< 0.0001
	C/NC	−0.0999	−0.2136	0.0139	0.0854
*for estimating the number of transitions (T*_*i*_*)*					
	Intercept	−1.4039	−1.5489	−1.2588	< 0.0001
	LENGTH	0.0001	0.0000	0.0003	0.0594
	DEPTH	0.0139	0.0119	0.0158	< 0.0001
	C/NC	−0.0398	−0.1683	0.0886	0.5435
*for estimating the number of transversions (T*_*v*_*)*					
	Intercept	−2.0007	−2.1868	−1.8147	< 0 .0001
	LENGTH	0.0003	0.0001	0.0005	0.0006
	DEPTH	0.0123	0.0099	0.0147	< 0.0001
	C/NC	−0.1883	−0.3539	−0.0226	0.0259

Estimates of variables from best fitting candidate model predicting the frequency of SNPs, transitions, and transversions in contigs (depth of 10 reads or more; length of 501 bp or longer).

^a^ LENGTH and DEPTH are the length and the depth of contigs, respectively, and C/NC is a dummy variable for the type of transcript (protein-coding (coded as 1) vs non-coding transcript (coded as 0)).

## Discussion

We used 454 pyrosequencing to generate 190 Mbp of tiger salamander transcriptome sequences that contained 2,980 putative SNPs. These high quality candidate SNPs, identified using strict criteria, can be converted into genetic markers for studies of natural selection, genomic organization, and allele-specific expression [14,32-34]. We used these data to develop new models for the estimation of SNP, *T*_*i*_, and *T*_*v*_ frequency; we then compared these estimates in coding and non-coding transcripts as discussed below.

### Modeling frequency estimates of SNP, *T*_*i*_, and *T*_*v*_

A number of recent studies have estimated SNP frequency using high-throughput sequencing technology by calculating the mean number of SNPs per unit sequence length. For example, Vera *et al.*[14] estimated a frequency of 6.7 SNPs/kb in butterflies, Külheim *et al.*[9] estimated 27.1 and 38.3 SNPs/kb in exons and introns, respectively, in eucalyptus, and Renaut *et al.*[7] found SNP densities of >20 SNPs/kb in genes for DNA transposition, mitotic spindle organization, and biogenesis. Such findings have provided broad perspective on SNP distribution across diverse species, but all of these studies considered only the effect of sequence length variation; *i.e.*, they estimated SNP frequencies per unit length without regard to the effect of variation in contig depth. Not unexpectedly, our data show that SNP frequency estimates are strongly and positively associated with contig length and with contig depth. Furthermore, our analyses suggest that polymorphism frequency estimates based on contig length-but-not-depth produce biased results; SNPs, *T*_*i*_, and *T*_*v*_ were more abundant in longer contigs assembled with more sequence reads. This finding suggests that both length and depth need to be considered for estimating the frequency of polymorphisms from next-generation sequence data.

A multi-model inference framework is informative for comparing the relative importance of the variables in our models. Our most robust model of frequency estimation was the global model that included contig length, depth, and transcript type. Importantly, contig depth is a more important variable than contig length when estimating SNP frequencies. This is clear from the Akaike weight (*w*_*i*_) of the models, because *w*_*i*_ is best interpreted as the relative likelihood or probability of the model [31]. Consider that our global model of SNP frequency estimation was only 9.4 times more likely than the next best model (contig depth and transcript type, but no length). In stark contrast, the global model was 7.5 × 10^55^ times more likely than the model of length and transcript type (*i.e.*, no depth; Table 2). In the context of massively parallel sequencing, this suggests that SNP frequency estimation by simply correcting for contig length is inadequate. Our results support the idea that SNP frequency increases with contig length and depth, but that contig depth is a much more meaningful factor in SNP discovery.

### Comparison of SNP frequency between protein-coding and non-coding transcripts

Our modeling approach can be used as a tool for inferring the intensity of selection on different categories of genes or genomic regions by comparing the SNP frequency among categories. Similar approaches have been developed for nucleotide diversity parameters such as beta (β) [35]. We studied the distribution of SNPs with regard to the protein-coding status of an expressed transcript, but our approach should prove informative in other comparisons across genomic categories (*e.g.*, exons *versus* introns, class I *versus* class II transposable elements, *etc.*).

In the current analyses, we included the dummy variable C/NC in all candidate models to test for differences in SNP frequencies between protein-coding and non-coding transcripts. In the long dataset with 10 or more reads we found a negative coefficient of C/NC, implying SNP frequency in non-coding transcripts is higher than that in protein-coding transcripts. Our use of a poly-dT primer for cDNA synthesis may have biased our representation of the transcriptome, but a similar pattern has been reported in human genomic DNA. Zhao *et al.*[8] investigated the distribution of human SNPs and found that SNP frequency was higher in intronic or intergenic than in exonic regions. Our results support the hypothesis that reduced SNP frequency in protein-coding regions relative to non-protein-coding transcripts can be attributed to the selection pressure on functionally important protein-coding regions [15,16]. However, absolute SNP frequency values we report here should be viewed with some caution because our transcripts were sequenced from a pool of seven tiger salamanders and pooled contig depth does not directly correspond to the actual number of haplotypes [35]. Although we cannot estimate standard nucleotide diversity parameters such as theta (θ) [36] from pooled transcripts from multiple individuals, SNP frequency by our modeling approach is still useful as a relative estimate to compare the selection pressure on different transcripts [35,37].

Our data indicate there is considerable variation in SNP frequency across transcribed sequences in *A. t. tigrinum*. These results are consistent with the observation that different classes of functional, non-coding RNAs (*e.g.*, microRNAs) evolve at different rates in humans and mice, with faster evolution in longer functional non-coding RNAs [18]. That said, our observed SNP frequency differences between coding and non-coding transcripts were subtle. There are several possible explanations for this. First, we relied on the CPC program [28] to parse protein-coding and non-protein-coding transcripts. CPC does so for each contig by evaluating the predicted ORF quality and the quality of the BLAST hit. This program generally performs well (accuracy of 92 ~ 96%; [28,29], but some transcripts remain ambiguous or are falsely allocated to the wrong category. In particular, categorical assignment (coding *vs.* non-coding) can be limited by the number of reference genomes available for comparison [29]. The tiger salamander is not a genomic model species (*e.g.*, no complete genome sequence is available) and as of yet has no close relatives with sequenced genomes, so this complicates the identification of orthologous sequences and protein homologs used in categorical (coding/non-coding) transcript assignments.

A second source of bias may be the short read sequences generated by next generation sequencing. For example, some of our sequences may be derived from short sections of an untranslated region (*e.g.*, 5’ or 3’ UTR) that occurs in a true protein-coding transcript. Such sequences will be erroneously categorized as non-coding transcripts due to poor ORF-related score, obfuscating SNP frequency differences between protein-coding and non-coding transcripts.

Finally, there may be true biological differences in SNP frequency between protein-coding and non-coding transcripts or within a transcript category—particularly when considering “long” transcripts. Recent analyses have revealed that even transcripts which do not encode proteins may have important functions and some of them are conserved to maintain functional domains and structures [17,38-40]. Many short non-coding transcripts such as microRNAs and small nucleolar RNAs are known to be highly conserved, functional non-coding RNAs across a diverse range of species [18]. In contrast, some long non-coding RNAs with a known function, such as *Xist* and *Air*, are poorly conserved across taxa [18,41]. This suggests rapid evolution of long non-coding RNAs despite their known functions (*e.g.*, involved in X-chromosome dosage compensation for *Xist* and silencing an imprinted gene at the *Igf2r* locus for *Air*) [42,43]. Such biological differences between long and short non-coding RNAs might be reflected in our “all” contig and “long” contig datasets. We found no difference in SNP frequency between coding *vs.* non-coding transcripts when all contigs were considered, but we did observe a trend of greater SNP density in non-coding transcripts compared to coding transcripts when only long contigs were considered. This observation provides indirect evidence that “long” non-coding transcripts have relatively fast rates of molecular evolution.

## Conclusions

Our transcriptome results revealed that estimates of polymorphism frequency are affected by both length and depth of contigs, such that simply dividing the total number of SNPs by the sequence length produces biased frequency estimates. In next-generation sequencing studies where reads vary dramatically among contigs, we propose that estimates of SNP frequency should be presented as *the standardized number of SNPs for a given contig length and depth* instead of the current “standard” of SNPs per unit length (*i.e.*, SNPs/kb). For example, polymorphism frequency might be reported as the number of SNPs in a 1 kb-long transcript with coverage depth of 10 reads. Even longer and deeper contigs will become more common as sequencing technology continues to advance (so it is currently fruitless to recommend a particular read depth), but authors should at least provide readers with information about contig depth as well as length when reporting SNP frequencies.

Our modeling approach revealed that long non-coding transcripts have marginally higher SNP frequencies than protein-coding transcripts. Future models that consider more variables may further refine our ability to estimate SNP frequency, so the absolute frequency values we report herein should be viewed with some caution. Nevertheless, the relative polymorphism frequency values reported herein support the hypothesis that long non-coding transcripts are less conserved than long protein-coding transcripts, perhaps as a function of selective constraints. We think that our modeling approach (*i.e.*, using multiple model construction and model selection approaches) can be a powerful tool for identifying selection on specific functional sequence groups by comparing the frequency and distribution of polymorphisms.

## Competing interests

The authors declare no conflict of interest.

## Authors’ contributions

SHE and JAD conceived and designed the experiments. SHE performed the experiments and analyzed the data. JAD contributed reagents/materials/analysis tools. SHE and JAD wrote the manuscript. All authors read and approved the final manuscript.

## Supplementary Material

Additional file 1 **Contigs and SNPs.** Information on 4,552 contigs with ≥10 sequence reads and ≥100 bp in length, and 2,980 SNPs found in the contigs. Click here for file

Additional file 2 **Supporting figures and tables.** A file containing additional data: Figure S1; Figure S2; Table S1; and Table S2. Click here for file
