# Supplementary material for: The effects of contig length and depth on the estimation of SNP frequencies, and the relative abundance of SNPs in protein-coding and non-coding transcripts of tiger salamanders (Ambystoma tigrinum)
Source: BMC Genomics. 2012 Jun 20;13:259. doi: 10.1186/1471-2164-13-259 (PMC3416719; doi:10.1186/1471-2164-13-259)
Supplement: Additional file 2 — Supporting figures and tables. A file containing additional data: Figure S1; Figure S2; Table S1; and Table S2. [file 1471-2164-13-259-S2.pdf]

Additional file 2 – Supporting figures and tables.

Table S1. Model selection among candidate regression models using negative binomial distribution predicting the frequency of SNPs, transitions, and transversions in contigs (depth of 10 reads or more; length of 100 bp or longer), using Akaike's Information Criteria (AIC).

Model	Parameters[a]	AIC	ΔAIC[b]	w_i[c]
for estimating the number of SNPs				
M1 (best, full model)	Intercept**, LENGTH**, DEPTH**, C/NC	9357.3	0.0	1
M2	Intercept**, DEPTH**, C/NC†	9390.6	33.3	5.8×10^{-8}
M3	Intercept**, LENGTH**, C/NC**	9583.2	225.9	8.7×10^{-50}
M4	Intercept**, C/NC**	9909.2	551.9	1.4×10^{-120}
for estimating the number of transitions (Ti)				
M1 (best, full model)	Intercept**, LENGTH**, DEPTH**, C/NC†	7296.8	0.0	1
M2	Intercept**, DEPTH**, C/NC*	7313.5	16.7	2.3×10^{-4}
M3	Intercept**, LENGTH**, C/NC**	7495.9	199.1	5.7×10^{-44}
M4	Intercept**, C/NC**	7741.8	445.0	2.4×10^{-97}
for estimating the number of transversions (Tv)				
M1 (best, full model)	Intercept**, LENGTH**, DEPTH**, C/NC	5071.0	0.0	1
M2	Intercept**, DEPTH**, C/NC	5109.3	38.3	4.9×10^{-9}
M3	Intercept**, LENGTH**, C/NC	5161.6	90.5	2.2×10^{-20}
M4	Intercept**, C/NC**	5369.7	298.7	1.4×10^{-65}

[a] LENGTH and DEPTH are the length and the depth of contigs, respectively, and C/NC is a dummy variable for the type of transcript (protein-coding (coded with 1) vs non-coding transcript (coded with 0). **, P < 0.01; *, P < 0.05; †, P < 0.1) variable in each model.

[b] ΔAIC is the difference between the AIC of the best fitting model and that of each model.

[c] w_i is Akaike weight of each model.

Table S2. Estimates of variables from best fitting candidate model predicting the frequency of SNPs, transitions, and transversions in contigs (depth of 10 reads or more; length of 100 bp or longer).

Parameter[a]	Estimate	95% confidence limits		*P*
for estimating the number of SNPs				
Intercept	-1.3056	-1.4092	-1.2020	< 0.0001
LENGTH	0.0004	0.0002	0.0005	< 0.0001
DEPTH	0.0142	0.0122	0.0161	< 0.0001
C/NC	0.0416	-0.0636	0.1467	0.4382
for estimating the number of transitions (Ti)				
Intercept	-1.6871	-1.8009	-1.5734	< 0.0001
LENGTH	0.0003	0.0002	0.0004	< 0.0001
DEPTH	0.0136	0.0117	0.0156	< 0.0001
C/NC	0.0990	-0.0188	0.2168	0.0997
for estimating the number of transversions (Tv)				
Intercept	-2.3380	-2.4857	-2.1903	< 0.0001
LENGTH	0.0005	0.0004	0.0007	< 0.0001
DEPTH	0.0115	0.0091	0.0139	< 0.0001
C/NC	-0.0576	-0.2112	0.0960	0.4625

[a] LENGTH and DEPTH are the length and the depth of contigs, respectively, and C/NC is a dummy variable for the type of transcript (protein-coding (coded as 1) vs non-coding transcript (coded as 0)).

A

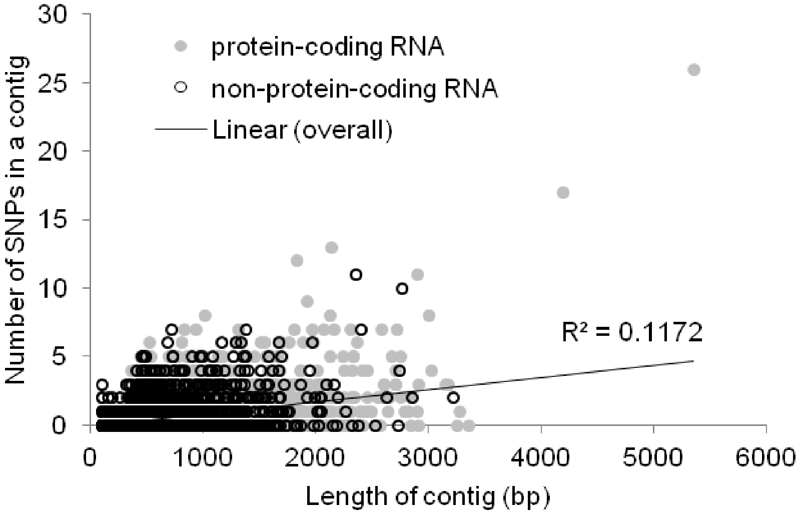

B

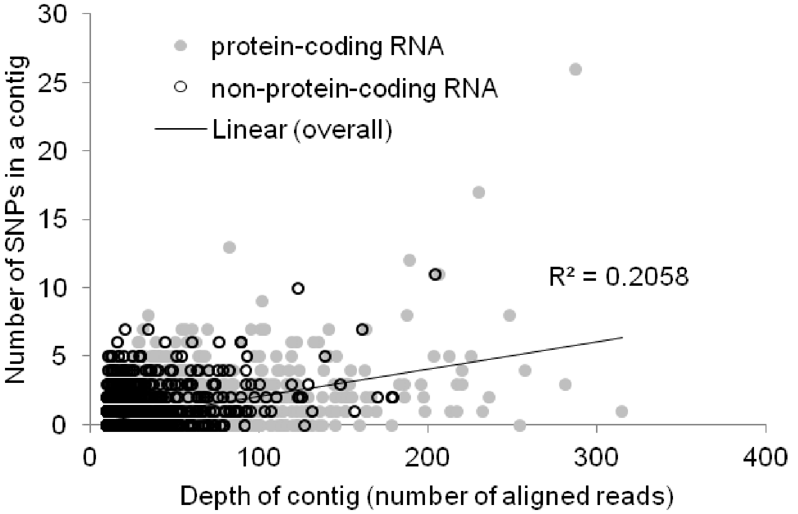

Figure S1. Positive correlation between the number of SNPs and both (A) length and (B) depth (both $P < 0.001$) of contigs with depth of 10 reads or more and length of 100 bp or longer (in (A), $R^2 = 0.13$ for protein-coding transcript and $R^2 = 0.08$ for non-coding transcript; in (B), $R^2 = 0.22$ for protein-coding transcript and $R^2 = 0.16$ for non-coding transcript).

A

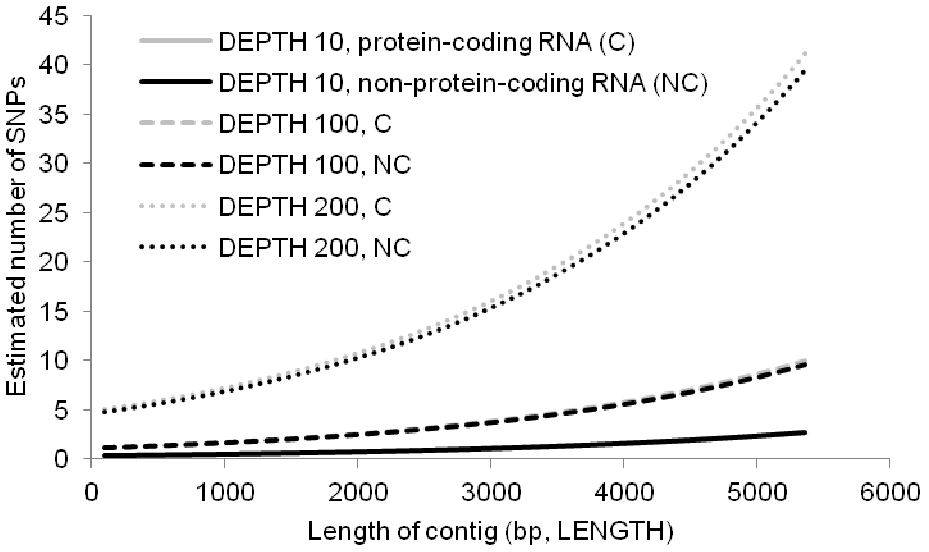

B

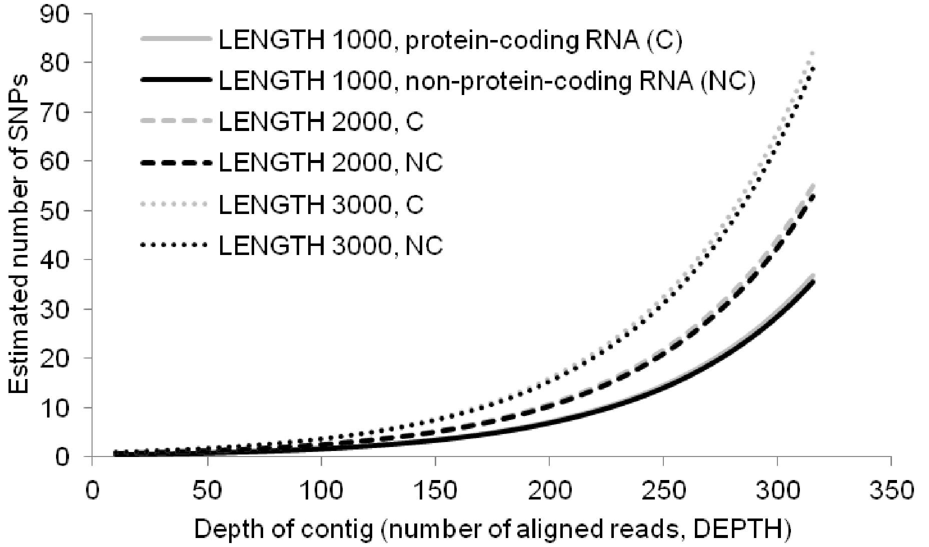

Figure S2. Estimates and comparison of the number of SNPs in protein-coding and non-coding transcripts based on various length and depth of contigs (depth of 10 reads or more; length of 100 bp or longer), using best fitting models (Table S2).
